# How does the New Cooperative Medical Scheme influence health service utilization? A study in two provinces in rural China

**DOI:** 10.1186/1472-6963-10-116

**Published:** 2010-05-10

**Authors:** Baorong Yu, Qingyue Meng, Charles Collins, Rachel Tolhurst, Shenglan Tang, Fei Yan, Lennart Bogg, Xiaoyun Liu

**Affiliations:** 1 Centre for Health Management and Policy, Shandong University, 44 Wenhua Xi Road, Jinan, 250012, Shandong, China; 2 Independent researcher, Liverpool, UK; 3 Liverpool School of Tropical Medicine, Pembroke Place, Liverpool, L3 5QA, UK; 4 Special Programme for Research and Training in Tropical Diseases (TDR), World Health Organization, Avenue Appia 20, 1211 Geneva 27, Switzerland; 5 School of Public Health, Fudan University. 138 Yi Xue Yuan Road, Shanghai, 200032, China; 6 Department of Public Health sciences, Karolinska Institute. SE-171 77 Stockholm, Sweden

## Abstract

**Background:**

Many countries are developing health financing mechanisms to pursue the goal of universal coverage. In China, a rural health insurance system entitled New Cooperative Medical Scheme (NCMS) is being developed since 2003. Although there is concern about whether the NCMS will influence the serious situation of inequity in health service utilization in rural China, there is only limited evidence available. This paper aims to assess the utilisation of outpatient and inpatient services among different income groups and provinces under NCMS in rural China.

**Methods:**

Using multistage sampling processes, a cross-sectional household survey including 6,147 rural households and 22,636 individuals, was conducted in six counties in Shandong and Ningxia Provinces, China. Chi-square test, Poisson regression and log-linear regression were applied to analyze the association between NCMS and the utilization of outpatient and inpatient services and the length of stay for inpatients. Qualitative methods including individual interview and focus group discussion were applied to explain and complement the findings from the household survey.

**Results:**

NCMS coverage was 95.9% in Shandong and 88.0% in Ningxia in 2006. NCMS membership had no significant association with outpatient service utilization regardless of income level and location.

Inpatient service utilization has increased for the high income group under NCMS, but for the middle and low income, the change was not significant. Compared with non-members, NCMS members from Ningxia used inpatient services more frequently, while members from Shandong had a longer stay in hospital.

High medical expenditure, low reimbursement rate and difference in NCMS policy design between regions were identified as the main reasons for the differences in health service utilization.

**Conclusions:**

Outpatient service utilization has not significantly changed under NCMS. Although utilization of inpatient service in general has increased under NCMS, people with high income tend to benefit more than the low income group. While providing financial protection against catastrophic medical expenditure is the principal focus of NCMS, this study recommends that outpatient services should be incorporated in future NCMS policy development. NCMS policy should also be more equity oriented to achieve its policy goal.

## Background

Many low and middle income countries are seeking ways to pursue the goal of universal coverage. To secure access to adequate health care for all at an affordable price, it will be necessary to increase the extent of prepayment and reduce the reliance on out-of-pocket payment [1,2]. Tax-based health financing and social health insurance, or a mix of them, are the most frequently used mechanisms for achieving this goal.

The Chinese government has been developing rural health insurance (Cooperative Medical Scheme, CMS) since the 1950s. By the late 1970s, approximately 90% of the rural population were covered by the CMS. The shift to a market economy and the demise of the rural communes in the 1980s was matched by the related decline in the CMS [3]. This left large sectors of the rural population uncovered by health insurance despite attempts by central and local governments to revive similar schemes in the 1990s [4]. In 2002 the State Council and the Central Committee of the Communist Party of China initiated the policy of the New Cooperative Medical Scheme (NCMS). This is a 'voluntary' and heavily subsidised scheme designed to reduce the financial burden of illness on the rural population. The Ministry of Health takes the overall responsibility to manage and supervise the scheme while the policy implementation responsibilities are decentralized to county level governments. The NCMS mainly covers inpatient services. It has been calculated that by the end of 2009, 95.3% of all counties and 91.5% (815 million) of the rural population would be covered by the NCMS [5].

Economic and health sector reform in China have been leading to increased inequity in health care, especially in rural areas [6,7]. Lack of health insurance, among many other factors, has been one of the main reasons contributing to this inequity [8-10]. While the expansion of NCMS coverage since 2003 is impressive, there is, however, concern over whether these coverage rates are matched by improvements in the utilisation of health services, and how the impact differs between different income groups [11-13]. In other words, although the NCMS aims in part to improve access to health services [14], it is not clear to date how the severe situation of inequity in access to health services has changed after the introduction of NCMS. An increasing body of studies published in Chinese have attempted to evaluate the effect of NCMS on health care utilization, but have presented contradictory findings in terms of outpatient and inpatient service utilization [15-17]. None of these studies have done a careful analysis on the confounding factors which may influence health service utilization. Studies published internationally have also analysed the NCMS [11,12], but these studies have mainly focused on the impact of NCMS on financial protection. We found only one internationally published study examining the impact of NCMS on health service utilization [13]. This is a quantitative study in 2005 comparing NCMS impact in 10 NCMS counties and 5 non-NCMS counties. It concluded that the NCMS has increased both outpatient and inpatient service utilization.

The aim of this paper is to assess the utilisation of health services under NCMS in two provinces of China. The study will compare the service utilization between NCMS members and non-members. Particular attention will be paid to the type of health services (outpatient and inpatient) and how the changes in health service utilization differ among different income groups and provinces. The importance of the paper is twofold. Firstly, it contributes to the limited evidence on health service utilization under NCMS, paying particular attention to utilisation differences between socio-economic groups. Secondly, the paper combines both quantitative and qualitative methods, the combination of which provides particularly rich explanatory data.

## Methods

### Study design and sampling

This study used a cross-sectional household survey to analyze the health service utilization among different income and regional groups under NCMS. Individual interviews, focus group discussions (FGD) and secondary data analysis were used to explore possible factors influencing health service utilization.

The study was conducted in Shandong and Ningxia Province, the choice of which allowed for analysis in different socio-economic and regional environments. Shandong is located in the more developed eastern coastal area and had a GDP per capita of 23,546 Yuan (about 3,139.5 US$) in 2006. Ningxia, a poor area in north western China, had a GDP per capita of 11,784 Yuan (about 1,571.2 US$) in 2006 and 36% of its population are ethnic minority groups. Three counties from each province were selected for the project. This selection was based on the following criteria: 1) all the counties had implemented NCMS, 2) the county governments were willing and able to collaborate with the research project, and 3) the three counties had different socio-economic development status representing the low, middle and high level of socio-economic development within the province.

The household survey used multistage sampling processes. Three townships in each county and six villages in each township were selected using similar criteria as the selection of counties. In each village, a random sample of 62 households for Shandong and 50 households for Ningxia was selected based on the household registration list. The calculation of sample size was based on the comparison of the expected utilization rate of inpatient services among different income groups. A total of 6,147 rural households and 22,636 individuals were included in the survey.

Many factors including health insurance status will influence people's utilization of health services. While some of the factors can be observed and measured in a questionnaire survey, some other factors are not easy to measure in such a quantitative way. We applied qualitative methods to understand other possible influencing factors and therefore to complement and explain findings from the household survey. In-depth individual interviews and FGDs were carried out with the rural residents, people suffering from catastrophic illness, policy makers (government officers from health department, civil affairs department, financial department, and township government), NCMS and health facility managers, and health providers from different levels of health facilities. To capture a range of experiences and views, the selection of residents included both male/female and members/non-members.

### Data collection

The field survey was carried out in May 2006 by researchers from Shandong and Fudan universities. A standard structured questionnaire was used for the household survey in all the six counties (Additional file 1). Questions related to this paper included: general demographic information of households and individuals (age, gender, education, marriage, annual household income and expenditure, and NCMS membership), perceived health needs (prevalence of self-reported sickness in the last 4 weeks and self-reported chronic disease in the last 12 months), health service utilization (utilization of outpatient service in the previous 4 weeks and inpatient service in the previous 12 months) and medical expenditure. After receiving training on the contents of the questionnaire and interview techniques, postgraduate students from both universities acted as the interviewers. Completed questionnaires were checked after the interview by research team members. Any missing questions and errors were corrected with the interviewers, or by returning to the household when necessary.

In-depth interviews and FGDs were carried out by senior qualitative researchers from Fudan University. Semi-structured topic guides were used in these interviews and FGDs. Questions related to this paper include: NCMS design in the study counties (health managers), reasons for using or not using health services (patients). All interviews were tape recorded with the informed consent from the respondents. In total, 26 groups of residents, 25 patients with catastrophic medical expenditure, 6 health managers from county health department, 13 NCMS managers, and 37 health providers were included in FGDs and interviews.

Relevant policy documents and secondary data on medical expenditure and fund allocation were also collected from routine statistics from the county NCMS office.

### Data analysis

Adverse selection may be an important concern in a voluntary insurance scheme like the NCMS. The nature of adverse selection is that people in poor health are more likely to join the scheme, while those in good health prefer not to join the scheme. Household income may also affect people's decision to join NCMS. As this paper compares the service utilization between members and non-members, there is a possible bias in data analysis due to adverse selection. Perceived health status as a proxy measure of need is one of the most important determinants of health service utilization [18,19]. Therefore, we first compared the prevalence rate of self-reported sickness for the previous 4 weeks, that of self-reported chronic disease for the previous 12 months and the average annual income between NCMS members and non-members.

Following this we compared health service utilization between NCMS members and non-members within each income group and each province respectively. Outpatient service utilization rate was defined as the number of interviewees using outpatient services in the last 4 weeks as a percentage of those who reported illness in the same time period. The research also used the inpatient admission rate for last 12 months and average Length of Stay (LOS) for inpatients as indicators to measure inpatient service utilization.

To define the household economic status, we used household income per capita to divide the study sample into three groups of equal size - the low, middle and high income groups. Annual income includes monetary income (from agriculture, salary and other sources) and income in kind (the monetary value of products such as grains, oil plants, vegetables, fruits, meat, and eggs, produced by the household and not sold) during the whole year of 2005.

Household survey data was analyzed using SPSS (16.0). Chi-square tests and multivariate regressions were applied for the analysis. Poisson regression is a conventional way of analyzing health service utilization data [20-22]. Data analysis shows that the mean values of frequency of outpatient visits and inpatient admissions (0.45 and 0.05 respectively) are close to their variance values (0.41 and 0.06). This shows that the indicators meet the requirement of Poison distribution. Therefore, Poisson regression was used to identify the association between NCMS and health service utilization. Since the LOS does not have a normal distribution, log-linear regression was used to identify the impact of NCMS and other factors of NCMS on LOS for inpatients service. The explanatory variables for multivariate regression included province, NCMS membership, gender, ethnicity, age, marriage status, educational status, occupation (farmer, non-farmer, teacher or student), income level, and self-perceived severity of sickness in the previous 4 weeks (only for analyzing the use of outpatient services), frequency of chronic diseases in the last 12 months (only for analyzing inpatient service utilization) and whether the individual had surgery (only for analyzing LOS). Since the use of multivariate regression was mainly to control confounding factors, we have not included the original regression results in this paper, but instead put the value of Relative Risk (RR), regression coefficient and 95% confidence intervals (CI) in the results.

Qualitative data were analysed using a 'framework approach' [23]. The analysis framework was developed based on the interview topic guides and emerging themes from the interviews. Each manuscript was repeatedly read and coded according to the framework. Coded sections were then retrieved and summarized into a matrix.

### Ethical approval

Ethical approval was obtained from the Research Ethics Committee at the Liverpool School of Tropical Medicine in UK and at the Center for Health Management and Policy of Shandong University in China.

## Results

This section introduces NCMS policy design in the two provinces, followed by a comparison of the health needs and income between members and non-members. The impact of NCMS on outpatient and inpatient service utilization and average LOS are then presented, together with possible factors affecting health service utilization.

### NCMS policy in two provinces

All six study counties started implementing NCMS between January and July 2005. By the time of this study, the household survey showed that NCMS coverage in the study counties was 95.9% in Shandong and 88.0% in Ningxia. The design and implementation of NCMS are largely decentralized to the county level health authority, although the counties operate within general national guidelines.

Table 1 sets out the basic structure and procedures of the NCMS system. Contributions were from individual members and subsidies from different levels of government. At the time of the research, the central government subsidies only covered the poor provinces in western and central China. Shandong, as an economically developed area, received no subsidy from central government. A family saving account (FSA) was set up in all three counties in Ningxia, and 8 Yuan of the individual contribution was allocated to the FSA, to cover outpatient service in township health centres. The FSA could be pooled for all household members and can be carried over to the next year, but there would be no further reimbursements for outpatient service beyond the FSA. For three counties in Shandong without FSAs, the NCMS covered 10-15% of drug expenditure for outpatient service in village clinics and township health centres, with a reimbursement ceiling of around 80 Yuan per member per year.

**Table 1:** The scheme design of NCMS in two provinces

	Ningxia	Shandong
Annual contribution per member (Yuan)	50	20-26
Central Government	20	0
Local government	20	10-16
Individual	10	10

Reimbursement rate for outpatient service (%)	8 Yuan per member per year from FSA	10-15

Deductible for inpatient service (Yuan):		
Township health centre	100	no
County hospital	200-300	1000 only in one county
higher hospital	500	no

Reimbursement rate for inpatient service (%)		
Township health centre	40-50	20-40
County hospital	35-45	15-45
higher hospital	15	5-10

Ceiling for total reimbursement per year for inpatient service (Yuan):	10,000	5,000

All remaining contributions were pooled into a fund at county level mainly to cover inpatient services. Members paid the full medical expenses for inpatient service at designated health facilities and then received reimbursements from the NCMS funds. The principles of a tiered reimbursement were used to set a higher reimbursement rate for a higher medical expenditure. For example, for a county hospital in one county in Shandong Province, the reimbursement rate was 15% for medical expenditure of less than 3,000 Yuan, 20% for medical expenditure of 3,000-5,000 Yuan, and 25% for expenditure of 5,000-8,000 Yuan. A reimbursement rate of 25% was set for medical expenditure of more than 200 Yuan at township health centres.

The reimbursement was governed by complex procedures designed to protect the financial stability of the funds. These stipulated deductibles, tiered reimbursement rates and ceilings. For inpatient services, a deductible was set in four of the six counties. Since government subsidies were less in Shandong, the reimbursement rates were lower compared with those in Ningxia. Reimbursement ceilings on inpatient care were set in all counties.

### Perceived health needs among NCMS members and non-members

The prevalence of self reported illness in the last 4 weeks and chronic disease in the last 12 months was analyzed to identify any possible difference in perceived health needs between NCMS members and non-members. Table 2 shows no significant differences of perceived health needs between the two groups (P > 0.05). Table 2 also shows that NCMS members and non-members had similar income level (P = 0.484).

**Table 2:** Economic status and health needs between members and non-members

	Non-NCMS	NCMS	P value
Annual income per capita (Yuan)	5,601	5,811	P = 0.484
Prevalence of illness in the last 4 weeks (%)	26.2% (455/1,739)	24.3% (5,076/20,889)	P = 0.082
Prevalence of chronic disease in last 12 months (%)	17.4% (303/1,739)	17.4% (3,640/20,889)	P = 0.999

### Impact of NCMS on outpatient service utilization

Chi-square test and Poisson regression were used to analyze the association between NCMS and outpatient service utilization in each income group (Table 3) and each province (Table 4).

**Table 3:** Health service utilization by NCMS membership and income group

	Low income group		Middle income group		High income group	
	
	Non-NCMS	NCMS	Non-NCMS	NCMS	Non-NCMS	NCMS
Outpatient service utilization rate (%)	39.4 (86/218)	38.5 (617/1603)	43.5 (54/124)	38.6 (641/1661)	33.6 (38/113)	38.8 (702/1812)
	RR: 1.13 95%CI: 0.90-1.42		RR: 0.95 95%CI: 0.73-1.24		RR: 1.24 95%CI: 0.91-1.69	

Admission rate (%)	3.9 (30/762)	5.2 (312/6005)	4.3 (22/506)	5.0 (348/7012)	2.5 (11/438)	4.4 (345/7872)
	RR: 1.39 95%CI: 0.97-2.00		RR: 1.16 95%CI: 0.76-1.76		RR: 1.84 95%CI: 1.03-3.28	

LOS (days)	7.00	10.05	7.86	7.97	9.36	11.32
	Coefficient: 0.080 95%CI: -0.045, 0.205		Coefficient: 0.257 95%CI: -0.051, 0.190		Coefficient: -0.012 95%CI: -0.224, 0.199	

RR and 95% CI for outpatient and inpatient service utilization are from Poisson regression results.

Coefficient and 95% CI for LOS are from log-linear regression results.

**Table 4:** Health service utilization by NCMS membership and region

	Shandong		Ningxia	
	
	Non-NCMS	NCMS	Non-NCMS	Non-NCMS
Outpatient service utilization rate (%)	38.8 (40/103)	37.7 (984/2,610)	39.2 (138/352)	39.6 (977/2,466)
	RR: 0.96 95%CI: 0.71-1.30		RR: 1.15 95%CI: 0.97-1.36	

Admission rate (%)	3.2 (14/437)	4.5 (509/11,347)	3.8 (49/1,302)	5.2 (496/9,542)
	RR: 1.47 95%CI: 0.87-2.51		RR: 1.35 95%CI: 1.02-1.78	

LOS (days)	4.50	8.57	8.63	11.00
	Coefficient: 0.210 95%CI: 0.044, 0.377		Coefficient: 0.016 95%CI: -0.075, 0.108	

RR and 95% CI for outpatient and inpatient service utilization are from Poisson regression results.

Coefficient and 95% CI for LOS are from log-linear regression results.

Table 3 shows sick NCMS members in the high income group used outpatient services slightly more than their non-member counterparts (38.8% vs. 33.6%), but the difference was not statistically significant. No differences were identified between NCMS members and non-members in the low and middle income groups (Table 3) and in either province (Table 4).

### Factors affecting outpatient service utilization

NCMS had a very low reimbursement level for outpatient services. In Shandong, the analysis of NCMS management data showed the reimbursement rate for outpatient was only 5.5% (13.5 Yuan out of 243.7 Yuan). The rate was even lower in Ningxia, only 2.2% (6.3 Yuan out of 274.3 Yuan). As a result, NCMS spent a low proportion of fund on outpatient services, namely between 25.9% and 30.0% in the six study counties in 2005. NCMS managers were well aware of this policy focus on inpatient service rather than outpatient service. As one NCMS manager reported during the interview: *'The fund for outpatient service in NCMS nearly has no function'*. This view was backed up by interviews with health care providers. Even poor families perceived that the low reimbursement proportion for outpatient service would not help their economic situation.

Qualitative findings show that high drug prices were another reason why people did not use more outpatient services under NCMS. Most NCMS members criticised the designated health institutions during FGDs, saying that they operated for profit as if they were private companies. They complained that drug prices under NCMS were too high in the NCMS designated health facilities and cheaper in private drug stores. NCMS members in Shandong province reported during FGDs: '*Even after reimbursement the (drug) price was still much higher in the designated hospitals than in the drug stores*. (We) *preferred to go directly to private drug stores.' *This image was confirmed by the health managers and health providers too. A county health manager pointed out that: '*...the quality of provided service was not as good and plus the higher drug price, these might lead members to lose faith and cause the failure of NCMS'*.

It was also acknowledged by members, NCMS managers and health providers that the reimbursement procedure was very complicated. The member had to show (at least) their NCMS certificate, identification card, and their bill for medical expenditure. Usually 4 to 5 signatures were required and, in some cases, the members had to visit the NCMS office several times. Most NCMS members reported they did not know all the detailed requirements and procedures. All of the above hardly constituted an incentive to use outpatient services for any income group.

### Impact of NCMS on inpatient service utilization

Table 3 indicates that the inpatient admission rate in the high income group was significantly higher for NCMS members (4.4%) than non-members (2.5%, RR = 1.84, 95% CI: 1.03-3.28). In the middle and low income groups, the admission rate of NCMS members was slightly higher than that of non-members, yet the difference was not statistically significant.

With respect to admission rates in the two provinces, Table 4 shows that in Ningxia province, NCMS members had a significantly higher admission rate (5.2%) than non-members (3.8%, RR: 1.35, 95% CI: 1.02-1.78), while in Shandong we did not identify a significant difference between the two groups.

Log-linear regression analysis showed no evidence that LOS was significantly different between NCMS members and non-members in any income groups (Table 3). However, NCMS members in Shandong province had a longer stay in hospital (8.57 days) than non-members (4.50 days, 95% CI of regression coefficient: 0.044-0.377), while in Ningxia Province, no significant difference was identified.

### Factors affecting inpatient service utilization

Firstly, this study confirms the relatively expensive nature of inpatient services in rural China, the high financial burden on households even for NCMS members, and the limited financial risk protection NCMS can provide to the rural population. Household survey data showed that the mean of medical expenditure was 3349.9 Yuan per inpatient admission. This accounted for an average of 87.8% of their annual net income. The average reimbursement rate for inpatient service was only 14.4% (479.7 Yuan). During FGDs, many NCMS members criticised the reimbursements for inpatient services as insignificant. Health managers and providers also reported that NCMS members still could not afford expensive treatment for catastrophic diseases due to the low NCMS reimbursement rates. One NCMS member from Shandong said in a FGD that *'the NCMS had no effect for real catastrophic disease at all, due to the expensive medical expenditure we would have to choose to die, and life was not worth that cost.' *Another villager from Ningxia reported that *'I heard from a neighbour that he visited the reimbursement office 3-4 times, but just got back 70 Yuan from the expenditure of 5000 Yuan*.' NCMS members also reported that some people had to bribe the staff in NCMS office to get their medical expenditure reimbursed. Villagers from both provinces also complained about the complicated administrative procedures.

Secondly, the practice in NCMS is that the members pay all medical expenses and then apply for reimbursement with a hospital receipt and other necessary documents of proof. The problem of the "pay first, claim later" model, particularly among the poor, was frequently raised during the interviews with health providers and managers. A physician reported a story about a meningitis patient being refused any treatment in a hospital just because the patients could not pay the full cost and the reimbursement of NCMS was low. Household survey data shows that among the people who refused doctors' recommendation to receive hospitalization services, the main reason for this was financial constraints; 91.0% (61/67), 65.1% (54/83) and 40.0% (14/35) cited this reason among the low, middle and high income groups respectively.

In explaining the difference in inpatient service utilization between the two provinces, two possible factors were explored. Firstly, health needs in terms of disease prevalence were different in Shandong and Ningxia. The prevalence of chronic diseases in the last 12 months is 18.8% in Ningxia and 16.1% in Shandong. The prevalence of self reported illness in the last 4 weeks is 26.0% in Ningxia and 23.0% in Shandong.

Secondly, these findings are consistent with a tendency towards a rational exploitation of the regulations by the members. Data analysis shows that medical expenditure was lower in Ningxia than in Shandong. For example, the mean medical expenditure per inpatient admission at county hospital was 2674.3 Yuan in Ningxia and 3635.0 Yuan in Shandong. At the same time members in Ningxia can receive a relatively higher reimbursement rate for inpatient service than those in Shandong, as shown in Table 1. This would provide the former with a greater incentive to seek inpatient care. In contrast, members from Shandong had less incentive to seek inpatient care, but once they did so they had an incentive to extend their LOS so as to gain from the higher reimbursement rates that came with higher expenditure. Another possible use of the regulations in Ningxia refers to the FSA and outpatient service. Once this fund was used up, members may well use the pooled fund for inpatient care for health problems that they might otherwise deal with through outpatient service.

## Discussion

### Limitations of the study

Adverse selection is usually an important concern in any voluntary health insurance scheme. If adverse selection exists, health insurance members may have higher health needs and health service utilization than those not covered by the insurance scheme. This will bring bias in examining impact on utilization. However, in the case of NCMS, the authors believe adverse selection is not a serious problem, for the following reasons.

Although NCMS is designed as a voluntary scheme, in the implementation process, high political will to increase the coverage has made it a quasi-voluntary scheme. As a result, the enrolment rate reached a very high level within 2-3 years of implementation. Furthermore, the design of NCMS has regulated that the participation unit should be household rather than individual to avoid the effect of adverse selection. Our analysis showed that the self-reported crude disease prevalence and income level among NCMS members and non-members are similar, indicating that adverse selection in NCMS is not significant. As explained in the methods section, the analysis of outpatient service utilization among those who reported acute illness and the inclusion of confounding factors in the multi-regression analysis can help overcome the possible confounding bias introduced by adverse selection.

Both the observed and many other unobserved differences between NCMS members and non-members may influence health service utilization. This study used multivariate regression analysis to analyze the association between the observed factors and service utilization. It also used a qualitative study to explore other possible influencing factors such as drug prices. Even so, we should excise caution in interpreting the causal effects relationship between NCMS and the changes in health service utilization.

### Rapid changes of NCMS policy since 2003

Since its establishment in 2003, the NCMS system has undergone rapid change, some of which has begun to address some of the problems identified in our findings. Firstly, the governments have increased subsidies to the NCMS fund. The central government began to subsidize not only the poorer provinces, but also poor areas in more developed eastern provinces such as Shandong. In 2008 the government subsidies reached 80 Yuan per person in Ningxia and 60 Yuan in Shandong [24].

Secondly, reimbursement procedures have also been simplified in most areas. NCMS reimbursement offices were set up in most designated health facilities. Patients can get reimbursement in these health facilities immediately after they have paid for their health services. It is reported that in the near future, patients will not need to pay the full cost in advance, but just need to pay the co-payment part [25]. This will further remove the financial barrier especially for the poor, which will in turn improve their access to health services.

### NCMS policy design: the balance between outpatient care and inpatient care

The results show that outpatient utilisation hardly changed under NCMS, regardless of income group and location. This confirms the NCMS policy focus on inpatient service rather than outpatients to achieve its principal goal of preventing catastrophic health expenditure. This finding is different from a previous study which found NCMS had increased both outpatient and inpatient services [13]. This difference however may well be due to different research sites (with different social development and disease profiles) used in the two research studies.

The balance of outpatient and inpatient services in benefit packages is a critical issue for policy makers to consider when introducing health insurance systems. Firstly, outpatient services should be an integral part of a comprehensive development of primary care. For many common health problems (especially some chronic diseases), outpatient services are more commonly used for effective and efficient interventions; therefore considerable expenses will occur in outpatient services [11,12]. Secondly, covering outpatient coverage from the beginning of a health insurance programme will provide the enrolees with more opportunities to experience its benefits and consequently helped to minimize drop outs, as is the case in South Korea [26]. The Chinese government has put a high priority on the extension of population coverage of NCMS, and started with a low benefit package. Within a few years' implementation NCMS has achieved a high coverage [5]. Since the premium level has been increasing from 30 Yuan per person in 2003 to 100 Yuan per person in 2008, the pool of NCMS funds is becoming larger. The benefit package could be extended incrementally. Health policy needs to work through the different options whereby outpatient service in the rural areas can play an integral role in primary health care development.

### Equity implication of NCMS

The recognised aim of the NCMS is to overcome the disease-poverty trap for the rural population. The scheme includes some pro poor elements, such as the government subsidy to the poor provinces.

The study shows the difference of inpatient services utilization between the high and low income group under NCMS. The high income group can afford the high co-payment level and is therefore more able to use inpatient services. For the low income group, however, even though the NCMS can provide some financial protection, the co-payment level is still too high for many of them to afford. Wagstaff et al found in their study that the rich have experienced a larger increase in inpatient care utilization although they also found that the poor have seen a larger increase in outpatient care utilization [13].

Another important feature of NCMS in relation to equity issues is its decentralised structure and the important roles given to the provincial and county levels. This may have its advantages in adapting health care to the specific circumstances of a region and contributing to greater local ownership. Although this research has not analysed these features, it has shown provincial differences in funding, NCMS structure and process in addition to utilisation. Two issues of equity are important in this analysis. Firstly, differences in utilisation patterns between provinces raise the issue of regional inequity and its association with decentralisation. Secondly, the central government subsidies are designed for equity in favouring resource allocation to the poorer provinces. This research, however, raises the important concern of who is benefiting from central subsidies. Our findings show that it is the rich, rather than the poor, who benefit the most from the central government subsidy in terms of utilisation. If equity is to be realised it is important that the means to ensure regional equity are made compatible with the means to ensure equity in healthcare between income groups within provinces and counties.

## Conclusions

This study of NCMS in six counties in the provinces of Ningxia and Shandong has shown the focus of the system on inpatient service and the important differences between different income groups and between provinces. The NCMS has increased inpatient service utilization, but there has been no significant change in outpatient service utilization. Among NCMS members, the high income group has increased inpatients service utilisation more than the low income group. Although NCMS has a certain underpinning in equity through its attempts to avoid the disease-poverty trap, a stronger and more positive strategy needs to be developed to make NCMS procedures more equity oriented. The importance of outpatient services should also be incorporated in the future NCMS policy development in rural China.

## Competing interests

The authors declare that they have no competing interests.

## Authors' contributions

All authors contributed to the design of the study. BY, QM, FY and XL collected and analyzed the research data. BY drafted the manuscript. CC, RT, ST, LB and XL made important contributions to the revision of the paper. All authors read and approved the final manuscript.

## Pre-publication history

The pre-publication history for this paper can be accessed here:

http://www.biomedcentral.com/1472-6963/10/116/prepub

## Supplementary Material

Additional file 1 **Questionnaire of household survey in China**. This additional file contains a household survey questionnaire used for RHINCAV project to evaluate the rural health insurance in six counties of China in 2006. Click here for file
