# Supplementary material for: How does the New Cooperative Medical Scheme influence health service utilization? A study in two provinces in rural China
Source: BMC Health Serv Res. 2010 May 10;10:116. doi: 10.1186/1472-6963-10-116 (PMC2907764; doi:10.1186/1472-6963-10-116)
Supplement: Additional file 1 — Questionnaire of household survey in China. This additional file contains a household survey questionnaire used for RHINCAV project to evaluate the rural health insurance in six counties of China in 2006. [file 1472-6963-10-116-S1.DOC]

Questionnaire of household survey in China

1. Name of the householder: household code: 2. Name of the interviewer: Interviewer code:

3. Name of the auditor: Auditor code： 4. Date of survey: (year) (month) (day)

5. Address of the household: Province County Township Village 6. Tel No.

**Table 1. General Information of Family Members (If some member is not available, other family member who knows him/her well should answer instead)**

| 1 | How many members are there in your family? |  |  |  |  |  |  |
| --- | --- | --- | --- | --- | --- | --- | --- |
| 1.1 | How many people lived in your house during the past six months?（including relatives, housekeepers, etc. who lived more than six months in the family） |  |  |  |  |  |  |
|  | Member code (01 is the code of the householder): | 01 | 02 | 03 | 04 | 05 | 06 |
| 2 | Name： |  |  |  |  |  |  |
| 3 | Relation with the householder: ①householder; ②Spouse; ③Children；④Grandchildren；⑤Parents; ⑥Grandparents; ⑦Siblings; ⑧others (please specify) |  |  |  |  |  |  |
| 4 | Is the questionnaire answered by himself/herself: ①Yes; ②No |  |  |  |  |  |  |
| 5 | Gender：①Male；②Female |  |  |  |  |  |  |
| 6 | Are you the Han Nationality: ①Yes; ②No (please specify) |  |  |  |  |  |  |
| 7 | Age |  |  |  |  |  |  |
| 8 | Marriage: ①Unmarried; ②Married; ③Divorced; ④Widowed |  |  |  |  |  |  |
| 9 | Education: ①Illiterate or semiliterate ②Preschoolers ③Elementary school ④Junior high school ⑤Senior high school ⑥Technical secondary school ⑦Junior college ⑧Bachelor degree or more advanced |  |  |  |  |  |  |
| 10 | Major job: ⑴Farmer; ⑵Factory worker; ⑶Village cadre; ⑷technical personnel; ⑸Teacher; ⑹General practitioner in village; ⑺Soldier; ⑻Business; ⑼Student; ⑽ Others (please specify) |  |  |  |  |  |  |
| 11 | Which health scheme do you belong to: ①None ②NCMS; ③Government employee scheme; ④Labor and semi-labor scheme; ⑤Basic insurance scheme for employees in urban areas; ⑥Commercial; ⑦Others (please specify) |  |  |  |  |  |  |
| 11.1 | If you have commercial insurance, how much do you pay as premium per year? |  |  |  |  |  |  |

**Table 2. Household General Information** (should be answered by the member who knows the household most well）

Household member code of the interviewee:

| 1 | How many meter squares is your living house? |  | 1.1 What type of building does your house belong to：①brick and wood；②concrete of brick；③clay；④stone；⑤others, please specify | | | | | | | | |  | |
| --- | --- | --- | --- | --- | --- | --- | --- | --- | --- | --- | --- | --- | --- |
| 2 | Does your household have following items (multi-choice):⑴TV ⑵Telephone ⑶Mobile phone ⑷Motorbike ⑸Farming machines ⑹Motor vehicle ⑺Washing machine ⑻refrigerator ⑼air conditioner ⑽VCD or DVD player | | | | |  | | | | | | | |
| 3 | How much was the total expenditure in your household in 2005（input +subsistence +other expenditures）? | | | | | | | |  | | | | |
| 3.1 | How much was the household productive expenditure (for farmer means the direct input into agriculture, i.e., expenditure for seeds, fertilizer, pesticide, annual cost of farming machines, etc.) in 2005? | | | | | | | |  | | | | |
| 3.2 | How much was the household subsistence expenditure in 2005? | | | | | | | |  | | | | |
| 3.2.1 | Therein, food expenditure: | | | | | | | |  | | | | |
| 3.2.2 | Clothing and other daily life item expenditure: | | | | | | | |  | | | | |
| 3.2.3 | Transportation and communication expenditure: | | | | | | | |  | | | | |
| 3.2.4 | Housing, electricity, water and fuel expenditure: | | | | | | | |  | | | | |
| 3.2.5 | Culture, education and entertainment expenditure: | | | | | | | |  | | | | |
| 3.2.6 | Drugs, health care, and medical supplies expenditure: | | |  | | | | | | | | | |
| 3.2.7 | Other consumption expenditures for living: | | | | | | | |  | | | | |
| 3.3 | How much was other expenditures (i.e. paying back debt and interest, premiums, gifts, penalty, kinds of fees forced from governments) in 2005? | | | | | | | |  | | | | |
| 4 | How much was the total revenue in the household during 2005? **（i.e. the sum up of items of 5.1）**？ | | | | | | | |  | | | | |
| 4.1 | Therein, agriculture revenue RMB，forest and cattle breeding, etc., revenue RMB，communication and transportation revenue RMB，wholesaling, retail, food trade revenue RMB，salary revenue RMB，part-time job revenue RMB，government subsidy and donation income RMB，revenue of other sources RMB。 | | | | | | | |  | | | | |
| 4.2 | What was the value (in local price) of grain, oil plant, vegetable, fruit, meat, eggetc. produced by your family but not sold value in 2005? | | | | | | | |  | | | | |
| 5 | If the household needs 7,000 RMB urgently, how will you collect the money: ①Use the bank deposit ②Borrow money from anyone. If ② is selected, how much should you borrow? | | | | | | | |  | | | | |
| 6 | Does the household have any debt or loan? ①Yes ②No ③No answer | | | | | | | |  | | | | |
| 6.1 | If has, how much is it? | | | | | | | |  | | | | |
| 6.2 | The reason for the debt is：①education; ②disease; ③marriage; ④building a new house; ⑤investment; ⑥others (please specify) | | | | | | | |  | | | | |
| 7 | Is your household assigned as poverty household in local area? ⑴Yes ⑵No | | | | | | | |  | | | | |
| 7.1 | If yes, what is the main reason that put the household into poverty: ⑴have not enough paid employment ⑵bad nature conditions or disaster ⑶disease or injury ⑷education ⑸ marriage ⑹others (please specify) | | | | | | | | | | | |  |
| 8 | How do you consider the household’s economy status in the village：① the best; ② good；③ moderate；④ inferior；⑤ the worst | | | | | | | |  | | | | |
| 9 | Which health facility is the nearest to your house: ①village clinic；②village drugstore；③township health center；④county hospital；⑤others (please specify) | | | | | |  | | | | | | |
| 9.1 | What is the distance in Kilometers from your house to the nearest health facility？ | | | | | |  | | | | | | |
| 10 | Is your household a member of the NCMS? ①No, never ②Used to be, but dropped out ③Yes | | | | | |  | | | | | | |
| 10.1 | If not a member, why: ①sickness will not be a burden to my family; ②No confidence in whether I can get health service; ③No confidence in insurance fund administration; ④don’t think it will solve the problem of high burden of disease; ⑤too complicated and boring to apply for reimbursement; ⑥the NCMS can’t last long; ⑦too poor to pay the premium; ⑧others (please specify) **(skip to question 18)** | | | | | | | | | |  | | |
| 10.2 | If used to be, but dropped out later, why: ①sickness will not be a burden to my family; ②No confidence in whether I can get health service; ③No confidence in insurance fund administration; ④don’t think it will solve the problem of high burden of disease; ⑤too complicated and boring to apply for reimbursement; ⑥the NCMS can’t last long; ⑦too poor to pay the premium; ⑧others (please specify) | | | |  | | | | | | | | |
| 10.3 | If you joined the NCMS, the main reason if: ①solidarity among the scheme members; ②better access to health care; ③supported by government fund; ④the family suffers from high medical expenditure; ⑤It is required by the government; ⑥The premium is a minor money to the family; ⑦Other families involved; ⑧others (lease specify) | | | |  | | | | | | | | |
| 10.3.1 | If the household is a member of the NCMS, how long has the membership lasted? | | | | Years and months. | | | | | | | | |
| 11 | Since joining the NCMS, had anyone in the household get medical reimbursement? ⑴Yes, got reimbursement on inpatient cost. ⑵Yes, got reimbursement on outpatient cost. ⑶Yes, got reimbursement both on inpatient and outpatient cost. ⑷No, never. | | | | | | |  | | | | | |
| 12 | Since joining the NCMS, did you get health consultation or any other prevention care covered by the NCMS: ①Yes (please specify); ②No | | | | | | |  | | | | | |
| 13 | Knowledge on NCMS (multiple-choice):①deductible; ②co-payment rate for hospitalization (for different hospital level); ③ceiling; ④Which institutions are assigned for NCMS ⑤Subsidy from the central and local governments ⑥Co-payment rate for outpatient visit | | | | | | | | | | | |  |
| 14 | Could you get information on NCMS reimbursement through mass median timely? ⑴Yes, I can get it timely. ⑵I can get it, but not timely. ⑶I never get it. ⑷I don’t know. | | | | | |  | | | | | | |
| 15 | Are you satisfied with the NCMS: ⑴Yes, perfectly satisfied. ⑵Satisfied. ⑶Not very satisfied. ⑷Dissatisfied very much. ⑸Don’t know. | | | | | |  | | | | | | |
| 15.1 | When you were not satisfied with the NCMS, did ever you have the willingness to complain: ⑴Yes ⑵No ⑶ Don’t know. | | | | | |  | | | | | | |
| 15.2 | When you were not satisfied with the NCMS, what did you do: ⑴Complained to the management office.⑵Complained to the assigned health facility. ⑶Complained to village manager.⑷Complained to the government. ⑸Complained to the mass median.⑹Complained to acquaintances. ⑺Never complained. ⑻Others (specify please) | | | | | |  | | | | | | |
| 15.3 | Do you think your complains worked or not: ①Yes, it worked. ②No. ③Don’t know. | | | | | |  | | | | | | |
| 16 | Which part of the NCMS do you think need to revise (multi-choice): ①premium; ②deductible; ③co-payment rate; ④procedure of applying for reimbursement; ⑤fund administration; ⑥others (please specify) | | | | | | | | |  | | | |
| 16.1 | According to current scheme, how much do you think the premium should be (in RMB)? | | | | | | | | | |  | | |
| 17 | What is the main way that you got information or knowledge on the NCMS: ①Radio ②TV ③Newspaper ④Propaganda/Dissemination leaflet, manual, bulletin board, etc. ⑤ Others’ saying ⑥ Health facility. ⑦Internet ⑻ Others (specify please) | | | | | | | | | | | | |
| 18 | Do you think the NCMS should be entered voluntarily or compulsorily? ①Voluntarily; ②Compulsorily; ③Not sure. | | | | | |  | | | | | | |
| 18.1 | Will you choose the membership of NCMS next year? ①Yes; ②No; ③Not sure | | | | | |  | | | | | | |
| 19 | Where would you like to pay the premium: ①At the bank; ②At the village clinic; ③At the township health center; ④At the county insurance office; ⑤Collected at home by government; ⑥Collected by the village autonomous organization; ⑦deducted in advance from the reimbursement; ⑧others (please specify) | | | | | | | |  | | | | |
| 19.1 | How often would you like to pay the premium? ①Yearly ②Once per two years or more ③ Don’t know | | | | | | | |  | | | | |
| 20 | Since the insurance fund is limited, what service do you think should be a priority (single choice): ①Outpatient visit ②Hospitalization ③Catastrophic events ④Preventive care ⑤others (please specify) | | | | | |  | | | | | | |
| 21 | Which organization do you prefer to manage the NCMS: ①Autonomous farmer organization ② Current authority in the village ③Township government ④County government ⑤Commercial insurance company ⑥others (please specify) | | | | | |  | | | | | | |
| If there was any member suffered from a chronic disease or gave a childbirth during the past year, go to Table 3 please; If there was member fell in sick in the past 4 weeks, go to Table 4 please; If any member experienced a hospitalization during the past year, go to Table 5 please.; If none of the above 3 cases, finish the survey here. | | | | | | | | | | | | | |

**Table 3. Health Service Utilization for Chronic disease and childbirth during the past year**

(If some member is not available or isn’t able to answer, other household member who knows him/her well should answer instead)

|  | Member code: |  |  |  |  |  |  |
| --- | --- | --- | --- | --- | --- | --- | --- |
| 1 | Do you have any chronic disease in the past year? ⑴Yes ⑵No**（Skip to 7）** |  |  |  |  |  |  |
| 2 | Name or main symptom of the first chronic disease（If you have more than one chronic disease, fill in each column by one disease） |  |  |  |  |  |  |
| 2.1 | Where was it diagnosed? ①Village clinic; ②Private clinic; ③Township health center; ④County hospital; ⑤Prefecture hospital or more advanced; ⑥No diagnosis; ⑦others (please specify) |  |  |  |  |  |  |
| 2.2 | Did you get any treatments in the past year? ⑴Yes ⑵No **(Skip to 4）** |  |  |  |  |  |  |
| 2.2.1 | Did you get any treatments in village clinic?  （1）Yes，I got times of treatments.（2）No**（skip to 2.2.2）** |  |  |  |  |  |  |
| 2.2.1.1 | How much money did you spend in village clinic for this disease? |  |  |  |  |  |  |
| 2.2.1.2 | How much was covered by the NCMS? |  |  |  |  |  |  |
| 2.2.1.3 | Are you satisfied with the service: ⑴Yes ⑵No |  |  |  |  |  |  |
| 2.2.1.3.1 | If not, the main reason is: ⑴Staff were not kind ⑵Technical skill was poor ⑶The facility and circumstance were not well ⑷Provided unnecessary services (including drugs and examinations) ⑸Medical charge was unreasonable ⑹Medical expenditure was high ⑺They didn’t give credit ⑻The procedure was too complicated ⑼Waiting time was too long ⑽ Others (specify please) |  |  |  |  |  |  |
| 2.2.2 | Did you get any treatments in the township health center?  （1）Yes，I got times of treatments.（2）No**（skip to 2.2.3）** |  |  |  |  |  |  |
| 2.2.2.1 | How much money did you spend in township health center for this disease? |  |  |  |  |  |  |
| 2.2.2.2 | How much was covered by the NCMS? |  |  |  |  |  |  |
| 2.2.2.3 | Are you satisfied with the service: ⑴Yes ⑵No |  |  |  |  |  |  |
| 2.2.2.3.1 | If not, the main reason is: ⑴Staff were not kind ⑵Technical skill was poor ⑶The facility and circumstance were not well ⑷Provided unnecessary services (including drugs and examinations) ⑸Medical charge was unreasonable ⑹Medical expenditure was high ⑺The procedure was too complicated ⑻Waiting time was too long ⑼ Others (specify please) |  |  |  |  |  |  |
| 2.2.3 | Did you get any treatments in the county hospital or more advanced hospital? （1）Yes，I got times of treatments.（2）No **(skip to 2.3）** |  |  |  |  |  |  |
| 2.2.3.1 | How much did you spend in the county hospital or more advanced hospital for this disease? |  |  |  |  |  |  |
| 2.2.3.2 | How much was covered by the NCMS? |  |  |  |  |  |  |
| 2.2.3.3 | Are you satisfied with the service: ⑴Yes ⑵No |  |  |  |  |  |  |
| 2.2.3.3.1 | If not, the main reason is: ⑴Staff were not kind ⑵Technical skill was poor ⑶The facility and circumstance were not well ⑷Provided unnecessary services (including drugs and examinations) ⑸Medical charge was unreasonable ⑹Medical expenditure was high ⑺The procedure was too complicated ⑻Waiting time was too long ⑼ Others (specify please) |  |  |  |  |  |  |
| 2.3 | Did you ever buy drugs in a drugstore? ⑴Yes ⑵No **(Skip to 3)** |  |  |  |  |  |  |
| 2.3.1 | How much did you spend in the drugstore? |  |  |  |  |  |  |
| 3 | Did the medical cost bring any difficulty to the household life: ⑴No, never. ⑵Yes, a little bit. ⑶Yes, a significant difficulty. ⑷Yes, it was a catastrophic event. |  |  |  |  |  |  |
| 4 | What is the main reason that you didn’t get any treatment? ⑴Did not perceive illness as severe enough to need treatment ⑵Financial difficulties ⑶No time ⑷Traffic barrier ⑸Poor quality of health services ⑹Have no effective measures ⑺Others (please specify) |  |  |  |  |  |  |
| 5 | In the past year, how many times had you ever been diagnosed to be hospitalized by a physician, but you were not hospitalize? (if no, fill with 0) |  |  |  |  |  |  |
| 5.1 | The reason you weren’t hospitalize was: ⑴Did not perceive illness as severe enough. ⑵no time ⑶financial difficulty ⑷poor quality of hospital service ⑸no bed ⑹too far, lack of transport ⑺others (please specify) |  |  |  |  |  |  |
| 6 | Generally speaking, how do you feel about your health status: ⑴Perfectly well ⑵Good ⑶Moderate ⑷Not well ⑸ Very terrible |  |  |  |  |  |  |
| 7 | If there was a childbirth in the household last year, how many ante-natal care consultations did the mother get? (if never, fill in with 0) |  |  |  |  |  |  |
| 8 | Was the childbirth: ⑴ A eutocia ⑵ A dystocia ⑶ A cesaream birth |  |  |  |  |  |  |
| 9 | Where was the childbirth born: ⑴Village clinic ⑵Private clinic ⑶Township health center ⑷County hospital or more advanced. ⑸Maternal and children care facility ⑹Home ⑺on the road ⑻others |  |  |  |  |  |  |
| 9.1 | If the baby was born at home, not in hospital, why: ⑴Had no necessary to go to hospital ⑵too urgent and had no time to go to hospital ⑶Economic difficulty ⑷Traffic barrier ⑸Others |  |  |  |  |  |  |
| 9.2 | If the baby was born at home, how much (RMB) did you pay the midwife? |  |  |  |  |  |  |
| 10 | Did the mother get post-natal care? ⑴Yes ⑵ No ⑶ Don’t know |  |  |  |  |  |  |

**Table 4. Illness and health seeking behavior during last 4 weeks** (if the same household member suffered from more than one illness, fill in one separate column with each illness)

|  | Family member code: |  |  |  |  |  |  |
| --- | --- | --- | --- | --- | --- | --- | --- |
| 1 | Name or main symptom of the disease you suffered during last 4 weeks |  |  |  |  |  |  |
| **2** | The illness you suffered or are suffering is: ⑴Acute disease occurred in last 4 weeks ⑵Acute disease occurred 4 weeks ago and lasted into last 4 weeks ⑶Chronic disease lasted into last 4 weeks ⑷Chronic disease occurred 4 weeks ago and lasted into last 4 weeks |  |  |  |  |  |  |
| 3 | How many days did this illness last for last 4 weeks |  |  |  |  |  |  |
| 4 | How did you feel the severity of your illness: ⑴Slight ⑵Moderate ⑶Serious (4)Not sure |  |  |  |  |  |  |
| 4.1 | In last 4 weeks, how many days did you rest from work (or school) because of this illness |  |  |  |  |  |  |
| 4.2 | In last 4 weeks, how many days did you rest in bed because of this illness (days) |  |  |  |  |  |  |
| 4.3 | The number of days with fever during last 4 weeks |  |  |  |  |  |  |
| 5 | Since fell into this illness, what measures did you take: ⑴Self-cared ⑵Visited a doctor **(skip to 9)** ⑶Self-cared firstly, then visited a doctor ⑷ Visited a doctor firstly, then self-cared ⑸Didn’t take any measure |  |  |  |  |  |  |
| 5.1 | 3.4.1What is the main reason that you didn’t take any measure: ⑴Did not perceive illness as severe enough to need treatment ⑵Financial difficulties ⑶No time ⑷Traffic barrier ⑸Poor quality of health services ⑹Have no effective measures ⑺Others (please specify) |  |  |  |  |  |  |
| **The following is concerned to those got treatments. Those who didn’t get any treatments finish Table 4.** | | | | | | | |
| 6 | Did you buy drugs in a drugstore: ⑴Yes ⑵No |  |  |  |  |  |  |
| 6.1 | How much did you spend on drugs from the drugstore for this illness during last 4 weeks (RMB) |  |  |  |  |  |  |
| 7 | Did your family have some pre-prepared medicines for self-care: ⑴Yes ⑵No |  |  |  |  |  |  |
| 7.1 | What was the value of these drugs (RMB)? |  |  |  |  |  |  |
| 8 | Besides the above, did you follow any other self care practices: ⑴Yes (specify please) ⑵ No |  |  |  |  |  |  |
| 8.1 | If yes, what were their costs (RMB)？ |  |  |  |  |  |  |
| **9 Outpatient visit** (if some member visited a doctor more than once, fill in one separate column with each visit) | | | | | | | |
|  | Family member code: |  |  |  |  |  |  |
| 9.1 | Which facility did you visit: ①Drugstore; ②Village clinic; ③Private clinic; ④Township health center; ⑤County hospital; ⑥Prefecture hospital or more advanced ⑦Others (specify please) |  |  |  |  |  |  |
| 9.2 | Why did you chosen this facility: ①Close to my house ②Good quality of services ③Price is low ④More kind ⑤I know the doctor ⑥Recommended by someone ⑦Have no other choice ⑧Others (please specify) |  |  |  |  |  |  |
| 9.3 | Did you get a diagnosis there? ①Yes ②No |  |  |  |  |  |  |
| 9.4 | How many minutes did you spend traveling from your home to the medical institution? |  |  |  |  |  |  |
| 9.5 | How many minutes did you spend waiting there? |  |  |  |  |  |  |
| 9.6 | Did you get administration of intravenous fluid there: ①Yes；②No |  |  |  |  |  |  |
| 9.7 | How much money did you spend in travel, accommodation and food, bonus or present for treating the illness? |  |  |  |  |  |  |
| 9.8 | What was the total medical expenditure in this institution? |  |  |  |  |  |  |
| 9.8.1 | Therein, how much was covered by NCMS? |  |  |  |  |  |  |
| 9.8.1.1 | How do you think about the procedure of reimbursement: ⑴Perfectly good ⑵Good ⑶Not bad, not good ⑷Bad ⑸Very bad |  |  |  |  |  |  |
| 9.8.2 | Therein, how much was covered by other schemes? |  |  |  |  |  |  |
| 9.9 | After meeting the doctor, did you buy drugs from other facility or drugstore: ⑴Yes (2)No |  |  |  |  |  |  |
| 9.9.1 | How much did these drugs cost？ |  |  |  |  |  |  |
| 9.10 | Did the medical cost bring any difficulty to the household life: ⑴No, never. ⑵Yes, a little bit. ⑶Yes, a significant difficulty. ⑷Yes, a catastrophic event. |  |  |  |  |  |  |
| 9.11 | Did the treatment: ①have no effect ②slight improvement ③great improvement ④Cured |  |  |  |  |  |  |
| 9.12 | How did you think about waiting time?⑴Too long; ⑵Acceptable; ⑶Quick |  |  |  |  |  |  |
| 9.13 | How did you think about the procedures to get treatment? ⑴Complicated; ⑵Acceptable; ⑶Simple |  |  |  |  |  |  |
| 9.14 | How did you think about the facility infrastructure? ⑴Poor; ⑵Average; ⑶Good |  |  |  |  |  |  |
| 9.15 | How did you think about the attitude of the health staff? ⑴Poor; ⑵Fair; ⑶Good |  |  |  |  |  |  |
| 9.16 | Were you diagnosed to be hospitalized, but you rejected to do so? ⑴Yes ⑵No |  |  |  |  |  |  |
| 9.16.1 | If you refused the inpatient service, why (single-choice): ⑴Did not perceive illness as severe enough. ⑵no time ⑶financial difficulty ⑷poor quality of hospital service ⑸no bed ⑹too far, lack of transport ⑺others (please specify) |  |  |  |  |  |  |

**Table 5. Hospitalization in the past 12 months（if the patient was hospitalized several times, fill one column for each hospitalization）**

| Family member code | |  |  |  |  |  |  |
| --- | --- | --- | --- | --- | --- | --- | --- |
| 1 | The reason you were hospitalized: ⑴Disease ⑵Injury or poisoning ⑶Rehabilitation ⑷Family planning ⑸Childbirth (6)Others (specify please) |  |  |  |  |  |  |
| 2 | Name of disease that caused your hospitalization: |  |  |  |  |  |  |
| 3 | What was the date when you were hospitalized? ( month/ year) | / | / | / | / | / | / |
| 4 | Where were you hospitalized? ⑴Township health center ⑵County hospital ⑶Prefecture hospital or more advanced ⑷Military hospital ⑸Others (specify) |  |  |  |  |  |  |
| 4.1 | Why did you select that institution? (1)Close to my home (2)Low price (3)Good quality (4)It is assigned (5)Have acquaintance (6)Have reliable doctors (7)Staff are kind （8）Recommended by someone（9）Others (specify please) |  |  |  |  |  |  |
| 5 | Had you received any surgery during this hospitalization? (1)Yes (2)No |  |  |  |  |  |  |
| 6 | What is the length of stay in hospital (days)? |  |  |  |  |  |  |
| 7 | The reason that you left hospital is: (1)Cured and under the request of the doctor (2)Not cured, but the doctor requested to leave (3)I requested to leave (4)Other reasons (specify please) |  |  |  |  |  |  |
| 7.1 | **If you requested to leave, why**? (1)I suffered from this disease too long and it seemed not better at all. (2)Financial embarrassment (3)Hospital environment (4)Staff were not kind (5)Others |  |  |  |  |  |  |
| 8 | What was the total medical expenditure (hospitalization, drugs, supplies, etc.) for this hospitalization? |  |  |  |  |  |  |
| 8.1 | During the hospitalization, did the physician ever tell definitely you which drugs and examinations were covered by the NCMS: ⑴Yes ⑵ No ⑶ Told me some items |  |  |  |  |  |  |
| 8.2 | How much had been covered by NCMS? |  |  |  |  |  |  |
| 8.2.1 | How do you think about the procedure of reimbursement: ⑴Perfectly good ⑵Good ⑶Not bad, not good ⑷Bad ⑸Very bad ⑹Not sure |  |  |  |  |  |  |
| 8.3 | How much had been covered by other schemes? |  |  |  |  |  |  |
| 9 | How much was spent on traveling and food for yourself and carers? |  |  |  |  |  |  |
| 10 | Did the medical cost bring any difficulty to household life: ⑴No, never. ⑵Yes, a little bit. ⑶Yes, a significant difficulty. ⑷Yes, a catastrophic event. |  |  |  |  |  |  |
| 11 | Did the treatment: ①have no effect ②slight improvement ③great improvement ④Cured |  |  |  |  |  |  |
| 12 | How did you think about waiting time?⑴Too long; ⑵Acceptable; ⑶Quick |  |  |  |  |  |  |
| 13 | How did you think about the procedures to get treatment? ⑴Complicated; ⑵Acceptable; ⑶Simple |  |  |  |  |  |  |
| 14 | How did you think about the facility infrastructure? ⑴Poor; ⑵Average; ⑶Good |  |  |  |  |  |  |
| 15 | How did you think about the attitude of the health staff? ⑴Poor; ⑵Fair; ⑶Good |  |  |  |  |  |  |
